# Age-dependent alterations of TRPA1 and urocortin 1 signaling in the Edinger–Westphal nucleus in a mouse model of Alzheimer’s disease

**DOI:** 10.1038/s41598-026-44022-5

**Published:** 2026-03-24

**Authors:** Antónia Petra Prókay, János Konkoly, Viktória Kormos, Tünde Biró-Sütő, Gábor Kriszta, Helga Panna Szentmártoni, Gergely Berta, Balázs Gaszner, Dóra Zelena, Erika Pintér

**Affiliations:** 1 Department of Pharmacology and Pharmacotherapy, Medical School, University of Pécs, Pécs, Hungary; 2 Department of Pharmacology, Faculty of Pharmacy, University of Pécs, Pécs, Hungary; 3 Pharmacology and Pharmaceutical Sciences Doctoral School, Medical School, University of Pécs, Pécs, Hungary; 4 Department of Medical Biology, Medical School, University of Pécs, Pécs, Hungary; 5 Department of Anatomy, Medical School, University of Pécs, Pécs, Hungary; 6 Institute of Physiology, Medical School, University of Pécs, Pécs, Hungary

**Keywords:** Alzheimer’s disease, Dementia, Edinger–Westphal nucleus, TRPA1, Urocortin 1, Ageing, Neurology, Neuroscience

## Abstract

Cholinergic neurons of the preganglionic Edinger–Westphal nucleus (EW) are involved in Alzheimer’s disease (AD), however, the role of urocortin 1 (UCN1) positive peptidergic neurons of the centrally projecting EW (EWcp) remains unclear. EWcp cells exclusively express transient receptor potential ankyrin 1 (TRPA1) ion channels, implicated in neurodegenerative disorders. We hypothesized that the EWcp/UCN1*/Trpa1* neurons may be involved in AD-related pathologies. Age-dependent (2, 6, 9, 12 and 18 months) *Trpa1* mRNA expression (RNAscope in situ hybridization) and UCN1 peptide (immunostaining) content of the EWcp were examined in male triple transgenic mouse (3xTg-AD) model of AD. ^1^H-MRI spectroscopy was performed in the hippocampus at 6, 12 and 18 months to evaluate the taurine and N-acetylaspartate levels, metabolites reflecting neuroprotection and neuronal integrity as AD prognostic markers. *Trpa1* expression was lower in 2 and 6-months-old 3xTg-AD than controls. Later the genotype differences disappeared due to the progressive, age-related reduction of *Trpa1* mRNA transcripts in the controls. In contrast, the *Trpa1* expression of transgenic mice remained persistently low. Similarly, the UCN1 peptide content was also lower in the 2 and 6-months-old 3xTg-AD compared to controls. However, UCN1 level increased with age, which was more pronounced in 3xTg-AD than controls abolishing the genotype differences. Age-dependent decrease in taurine level was detected in transgenic animals leading to significantly lower taurine/creatine ratio in 12 and 18-months-old 3xTg-AD animals compared to the controls. This age-related dynamics of *Trpa1* and UCN1 expression of 3xTg-AD mice suggests that altered UCN1 signaling may contribute to AD-associated mood disorders and memory decline.

## Introduction

Dementia is a progressive neurodegenerative condition characterized by a decline in memory, cognitive and functional abilities affecting globally ca. 47 million individuals^1^. Several pathologies (*e.g.* traumatic brain injury, cerebrovascular and metabolic disorders) may contribute to its process^2–5^; however, Alzheimer’s disease (AD) remains the leading reason accounting for 60–70% of dementia cases over 65^6^. Pathogenetic factors leading to neurodegeneration in AD are still not completely revealed, though the extracellular amyloid plaque deposition (amyloid β,Aβ-theory) inducing neuronal apoptosis^7^, as well as the intracellular accumulation of neurofibrillary tangles (tau-theory) resulting in disturbed axonal transport^8–10^ are common hallmarks observed in the disorder.

The damage of the basal forebrain cholinergic neurons is a well-known phenomenon upon AD^11–13^, and treatment strategies involve primarily the restoration of cholinergic neurotransmission^14,15^. However, less attention is paid to the role of other cholinergic areas.

The Edinger–Westphal nucleus (EW) is located in the midbrain and is composed of two main neuronal populations that significantly differ in their neurochemistry, connectivity and function. The neurons comprises the centrally projecting Edinger–Westphal nucleus (EWcp), made up by neurons of peptidergic neurochemical character. The majority of these cells express urocortin 1 (UCN1; marker of these neurons) and other pain-related neuropeptides. These peptidergic cells project to several brain areas and have been implicated in the regulation of stress adaptation, circadian rhythm, pain, food intake and alcohol consumption. Another group of the neurons is the preganglionic EW (EWpg), made up of parasympathetic cholinergic neurons (choline acetyltransferase is the marker of these neurons). This is classically considered as part of the oculomotor nuclear complex involved in the control of intraocular muscles for lens accommodation and pupillary light reflex^16,17^.

The damage of cholinergic neurons in the EWpg was described in the early stage of the disease^18^, leading to disturbed pupillomotor function^19–22^. On the other hand, the EWcp includes a neuronal population^17^ expressing mainly neuropeptides (*e.g*., urocortin 1; UCN1), though cells exhibiting cholinergic neurochemical characteristics may also be found here^23^. The EWcp plays an important role in stress adaptation and mood regulation^16^,moreover, it receives afferent fibers from the hippocampus modifying attention and cognition^24^. Interestingly, the neurodegeneration of EWcp contributes to the non-motor symptoms of Parkinson’s disease in a rat model^25^. The highest mRNA expression of the transient receptor potential ankyrin 1 (TRPA1) cation channel was observed in the EWcp with exclusive UCN1 co-localization both in mice and in humans^26^, where we also confirmed its functional activity by patch clamp technique^27^. Literature data suggests that TRPA1 contributes to AD pathology as the mediators released during neurodegeneration, inflammation or oxidative stress are able to activate this ion-channel^28–34^. The EWcp/UCN1/*Trpa1* neurons project to limbic regions implicated in AD pathogenesis (prefrontal cortex; PFC, amygdala)^35,36^, to hypothalamic nuclei (paraventricular, supraoptic nuclei and lateral hypothalamic area)^37^, and to certain brainstem areas (dorsal raphe nucleus,DRN as well^38^ regulating stress adaptation, thermoregulation, food intake and mood,each of them impaired in AD^39–42^. Importantly, some of these projection areas are involved in memory regulation as well^43–45^ suggesting a further relationship between EWcp and AD.

Hippocampus is the key component of limbic system playing an important role in memory^46,47^. Previous studies demonstrated that projection pathways from the hippocampus extend to the EWcp potentially influencing its functions^24,48,49^. The small size of the EWcp limits the neuroimaging analysis by MRI, thus we aimed to validate our model by focusing on the hippocampal metabolite levels. We assume that changes in the hippocampal metabolic processes may affect the EWcp function as well. Altered hippocampal metabolism might be an early sign of AD, which can be detected by N-acetylaspartate (NAA) and taurine ^1^H-MRI spectroscopy, two biomarkers reliable for characterizing brain metabolism. NAA serves as an indicator of neuronal viability and mitochondrial function, whose level are consistently reduced in AD, particularly in the hippocampus^50,51^. The decrease in NAA correlates with the deposition of Aβ-plaques and the formation of neurofibrillary tangles^52,53^ and also reflects the loss of neurons in the affected brain regions, such as the hippocampus and entorhinal cortex^50,51,54,55^, while its levels may remain unchanged in less involved areas *e.g.* in the cerebellum^53^. Taurine is involved in neurotransmitter modulation, calcium homeostasis, osmotic regulation, and antioxidant defense. The key pathological mechanisms of AD (*e.g*., oxidative stress, calcium dysregulation, and neuroinflammation) are associated with decreased taurine levels amplifying the neuronal damage. Animal studies demonstrated that taurine has neuroprotective effects in AD models by inhibiting Aβ-accumulation, tau hyperphosphorylation, mitigating oxidative damage, and improving cognitive performance^56–58^.

Here, we hypothesize an age-dependent decline in the EWcp/UCN1 neurons, which will be more pronounced in 3xTg-AD, compared to controls. Moreover, we assume that TRPA1 ion channels are crucial elements, as mediators released during neurodegenerative and neuroinflammatory processes may modulate the function of these neurons via TRPA1 activation. In order to explore the possible correlation with cognitive decline, we evaluated age-related changes in taurine and NAA levels in the hippocampus.

## Materials and methods

### Animals

Triple transgenic (3xTg-AD) male mice (B6;129-Tg (APP_Swe_, tauP301L)1Lfa Psen1tm1Mpm/Mmjax; #034830-JAX; local homozygous breeding at University of Pécs, Medical School, Institute of Physiology) were used as an in vivo model of AD. In transgenic animals, human genes known to be implicated in the pathogenesis of AD—amyloid precursor protein (APP), presenilin-1 (PSEN1), and tau-protein—are overexpressed. The age-dependent neuropathological changes (Aβ plaque deposition, neurofibrillary tangle formation, and synaptic dysfunction) imitating the progression of AD (Oddo et al. 2003) ^134^ support the relevance of this model. Animals were housed in the animal facility of the Department of Pharmacology and Pharmacotherapy, University of Pécs in a temperature and humidity controlled 12 h light–dark cycle environment (lights on at 6 a.m.) in standard polycarbonate cages (365 mm × 207 mm × 144 mm). Ad libitum standard rodent chow and tap water were provided for the animals. Four to six mice were housed in one cage. All experiments were approved by the Workplace Animal Welfare Committee of the University of Pécs (BA02/2000-89/2023) and performed according to the European Communities Council Directive recommendations for the care and use of laboratory animals (2010/63/EU). The authors complied with the ARRIVE guidelines.

### Experimental design

Experiment 1.: Intact 2, 6, 9, 12, and 18-month-old male 3xTg-AD and C57Bl/6 J mice (n = 4–6 per group) were compared. The *Trpa1* mRNA expression and the UCN1 density of the EWcp urocortinergic neurons were investigated by RNAscope in situ hybridization (ISH) combined with immunostaining.

Experiment 2.: Independent cohort of intact 6, 12, and 18-month-old male 3xTg-AD and C57Bl/6 J mice (n = 4 per group) were compared for the NAA and taurine metabolites in the hippocampus by MRI-spectroscopy. Originally, we aimed to study EW as well to reveal direct correlation, but the resolution of our MRI did not allow us to evaluate it.

### Perfusion, tissue sample collection

In Experiment 1. mice were euthanized with intraperitoneal (i.p.) injection of overdose urethane (2.4 g/kg). Transcardial perfusion was performed with 20 mL of ice-cold 0.1 M phosphate-buffered saline (PBS, pH 7.4), followed by 150 mL of 4% paraformaldehyde (PFA) solution. Brains were removed and postfixed in PFA at 4 °C for 72 h. Coronal sections of 30 µm thickness were prepared using a Leica VT1000S vibratome (Leica Biosystems, Wetzlar, Germany), and slices were stored in antifreeze solution (20% ethylene glycol, 30% glycerol, and 0.1 M sodium phosphate buffer) at − 20 °C. Samples containing the EWcp (Bregma − 2.92 to − 4.04 mm) were collected based on the stereotaxic coordinates of the mouse brain anatomy atlas (Paxinos and Franklin, 2001) ^135^.

### RNAscope in situ hybridization combined with immunofluorescence

RNAscope ISH was performed on coronal sections of EWcp to detect *Trpa1* mRNA. The pretreatment procedure was optimized for 30 µm vibratome-sectioned slices earlier^59^. Subsequent steps, including probe hybridization, sequential signal amplification and channel development, were carried out according to the RNAscope Multiplex Fluorescent Reagent Kit v.2 user manual (Advanced Cell Diagnostics, Newark, CA, USA). For hybridization, the following probes were used: mouse *Trpa1* (Cat No: 400211-C2), 3-plex positive control (Cat No: 320881) targeting RNA polymerase II subunit A (*Polr2a*; fluorescein), peptidylprolyl isomerase B (*Ppib*; Cyanine 3 (Cy3)), and ubiquitin C (*Ubc*; Cyanine 5 (Cy5)) mRNAs, moreover, 3-plex negative control (Cat No: 320871) (bacterial D-box binding PAR BZIP transcription factor (*dabP*)).

For the visualization of UCN1 peptide, immunofluorescence was performed after the RNAscope ISH procedure. Briefly, sections were incubated overnight at room temperature (RT) with recombinant rabbit anti-urocortin 1 antibody (Cat. No.: ab28503;^43,60,61^) diluted 1:10 000 in PBS containing 2% normal donkey serum (NDS) followed by 2 × 15 min washing in PBS. On the second day, sections were incubated with Alexa Fluor488-conjugated donkey anti-rabbit secondary antibody (Jackson ImmunoResearch Europe Ltd., Cambridgeshire, UK, Cat No: 711-545-152) diluted 1:500 in PBS containing 2% NDS for 3 h at RT followed by 2 × 15 min washing. Subsequently, 4′,6-diamidino-2-phenylindole (DAPI; Cat No: 323108, Advanced Cell Diagnostics, Newark, CA, USA) was applied to counterstain the nuclei. Finally, sections were mounted with ProLong Diamond Antifade Mountant (Thermo Fisher Scientific) and stored at − 20 °C until the confocal microscopy.

### Microscopy, imaging and morphometry

Olympus FluoView FV-1000 laser scanning confocal microscope and FluoView FV-1000S-IX81 image acquisition software system (Olympus Europa, Hamburg, Germany) were used for imaging. To eliminate false positive signal triggered by overlapping emission spectra, and to accurately quantify fluorescence signals, digital images were acquired using sequential scanning in analog mode for each fluorophore. During sequential scanning, the confocal aperture was set to 80 µm, with an optical thickness of 3.5 µm, using a 60 × oil immersion objective lens (NA: 1.65) and a resolution of 1024 × 1024 pixels. Excitation and emission spectra for the fluorophores were adjusted as specified by the FluoView software (DAPI—405 nm, fluorescein and Alexa Fluor488—488 nm, Cyanine 3 (Cy3)—543 nm, Cyanine5 (Cy5)—647 nm). Fluorescence signals with different emission spectra were assigned virtual colors by the software: blue for DAPI, green for fluorescein and Alexa Fluor488, red for Cy3, and white for Cy5.

Morphometric analyses were conducted using ImageJ software (version 1.52a, NIH, USA) on raw images. Measurements for both *Trpa1* mRNA and UCN1 peptide were performed on three slices per animal containing EWcp with 5–10 neurons per slice. For UCN1 peptide, the region of interest was manually marked in the cytoplasmic regions of neurons, excluding the nuclear area. Specific signal density (SSD) was expressed in arbitrary units (au). SSD values were calculated for 5–10 neurons per slice, averaged per slice, and the mean background density was subtracted from the mean signal density. The average, background corrected SSD values from three slices per animal were used to determine the mean SSD value for each mouse. To estimate the changes in *Trpa1* mRNA expression, the number of copies per cell was manually counted in 5–10 neurons per section, in three representative sections of EWcp per animal. UCN1 immunofluorescence was used to visualize cell borders, as well as distinguish between the neurons. We counted the number of *Trpa1* mRNA copies only in cells that were sectioned in the plane of the nucleus, thereby increasing standardization. These values were averaged as described above and subjected to the statistical assessment.

### MRI-spectroscopy

**Fig. 1:**
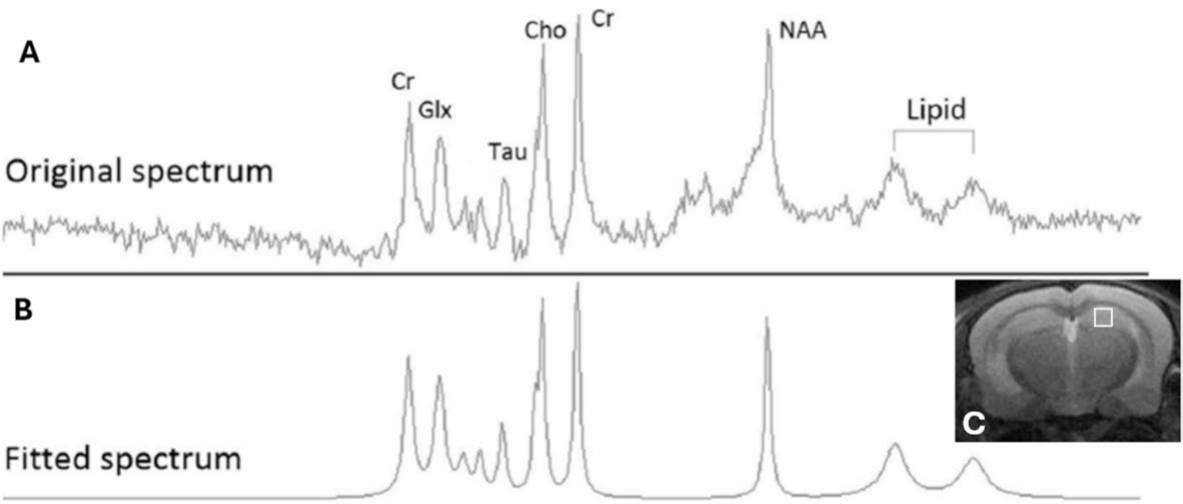
Representative images about the original raw (**A**) and fitted processed (**B**) spectrum, with major metabolites annotated (Cr: total creatine, Glx: glutamate + glutamine, Tau: taurine, Cho: choline, NAA: N-Acetylaspartate, Lipid: broadening lipid peaks). Representative T2-weighted MRI image, with the ROI demarcated (**C**).

In vivo ^1^H MRI-spectroscopy was performed on the hippocampus of mice by small animal MRI instrument (Bruker® PharmaScan® (4.7 T) Billerica, MA, USA). The T2-weighted scans were used to position the region of interest (ROI) for spectroscopy (based on anatomical structures) in the midbrain to cover the hippocampus^62^ (Fig. 1.). MRI-spectroscopy measurements were carried out under sevoflurane anesthesia (4% for induction and 1.5–3% for maintenance) in a 3:1 mixture of nitrous oxide and oxygen. Body temperature and respiration rate of the animals were continuously monitored. For voxel positioning, after B_0_ map acquisition, T2 RARE images were acquired in all three cross-sectional directions, and a 2.5 mm edge length cubic volume was assigned for localized shimming (Bruker Mapshim method) and spectroscopy (PRESS sequence, measurement time circa 20 min), according to the anatomical structures. Raw data were evaluated using Mnova software package (Mestrelab Research, S.L.U. Avenida de Barcelona 7-15706 Santiago de Compostela, SPAIN). The NAA and taurine metabolites were measured. Peak extraction and assignation followed by integration were performed. The original raw spectrum was displayed, with major metabolites annotated (total creatine, glutamate + glutamine, taurine, choline, NAA and lipid: broadening lipid peaks (Fig. 1A, B). Figure 1C displays a representative T2-weighted MRI image, with the ROI demarcated. No external reference was used during the measurement. Since it is known from the literature that creatine levels remain constant in the brain during aging, all peaks were normalized to individual creatine levels and expressed as percentages^63,64^.

### Statistical analysis

Statistical analysis was carried out using Statistica software (version 13.5.0). Data were expressed as mean ± standard error of the mean (SEM). Normality of distribution was assessed using the Shapiro–Wilk test (Shapiro and Wilk, 1965) ^136^, and homogeneity of variance was evaluated with Bartlett’s Chi-square test (Snedecor and Cochran, 1989) ^137^. The main effects were studied by two-way analysis of variance (ANOVA, variables: genotype and age), followed by Tukey’s post hoc test (Tukey^65^). *p* < 0.05 was considered statistically significant.

## Results

### Significant age-dependent changes were observed in the UCN1 peptide content of the EWcp in male 3xTg-AD model

**Fig. 2:**
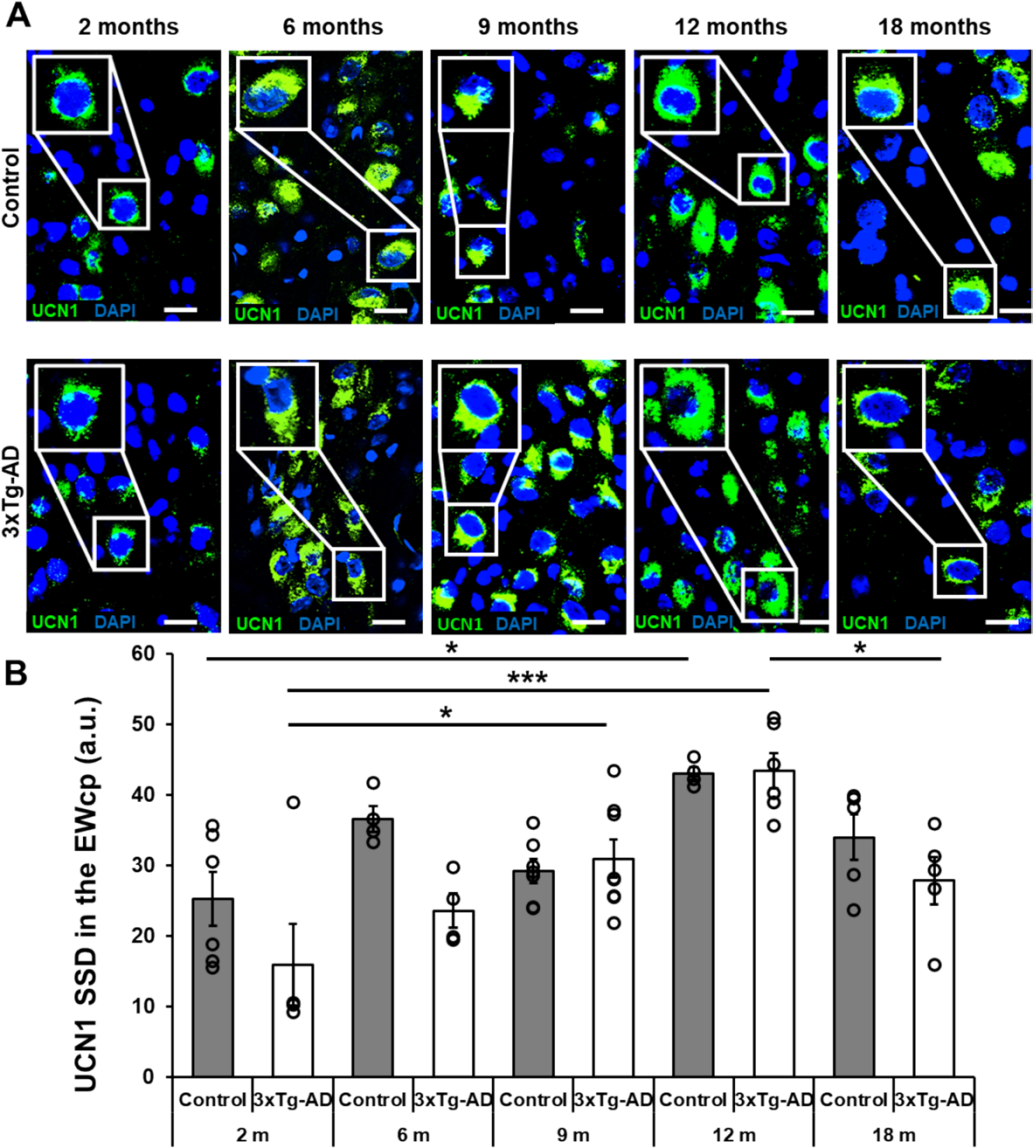
Representative confocal images of the urocortin 1 (UCN1) peptide (green) content in the centrally projecting Edinger–Westphal nucleus (EWcp) of control and triple transgenic (3xTg-AD) mice. Sections were counterstained with 4′,6-diamidino-2-phenylindole (DAPI) (blue) for nuclei. Scale bars: 25 μm. Age-dependent changes in the specific signal density (SSD) of UCN1 in the EWcp neurons of control and 3xTg-AD mice (**A**). Two-way ANOVA followed by Tukey’s post hoc test; *p < 0.05 and ***p < 0.001; n = 4–6. a.u.: arbitrary unit (**B**).

Statistical analysis revealed gradual increase in the UCN1 expression in both genotypes until 12 months of age compared to the younger counterparts with a significant main effect of age (F_age_ (4,44) = 12.28; *p* < 0.0001). Interestingly, this enhancement was more expressed in the 3xTg-AD animals (post hoc comparison p_2month/6 month_ < 0.35, p_2month/9 month_ < 0.99, p_2month/12 month_ < 0.02 in controls, while p_2month/6 month_ < 0.86, p_2month/9 month_ < 0.03, p_2month/12 month_ < 0.0002 in transgenic mice), followed by a decrease in peptide content of transgenic animals by the age of 18 months (p_12month/18 month_ < 0.04). Although ANOVA revealed significant main effect of genotype as well (F_genotype_ (1,44) = 6.67; *p* < 0.02) with lower levels in 3xTg-AD compared to controls, but post hoc comparison revealed only a weak tendency between the 6-months-old control and transgenic animals (*p* < 0.30) which was completely abolished in the older age groups (post hoc test *p* > 0.94 in each comparison from 9 month of age) with a strong tendency of interaction between the two variables (F_interaction_ (4,44) = 1.95; *p* < 0.12) (Fig. 2).

### Lower *Trpa1* mRNA expression was observed in male 3xTg-AD AD model with more pronounced difference from controls during younger age

**Fig. 3:**
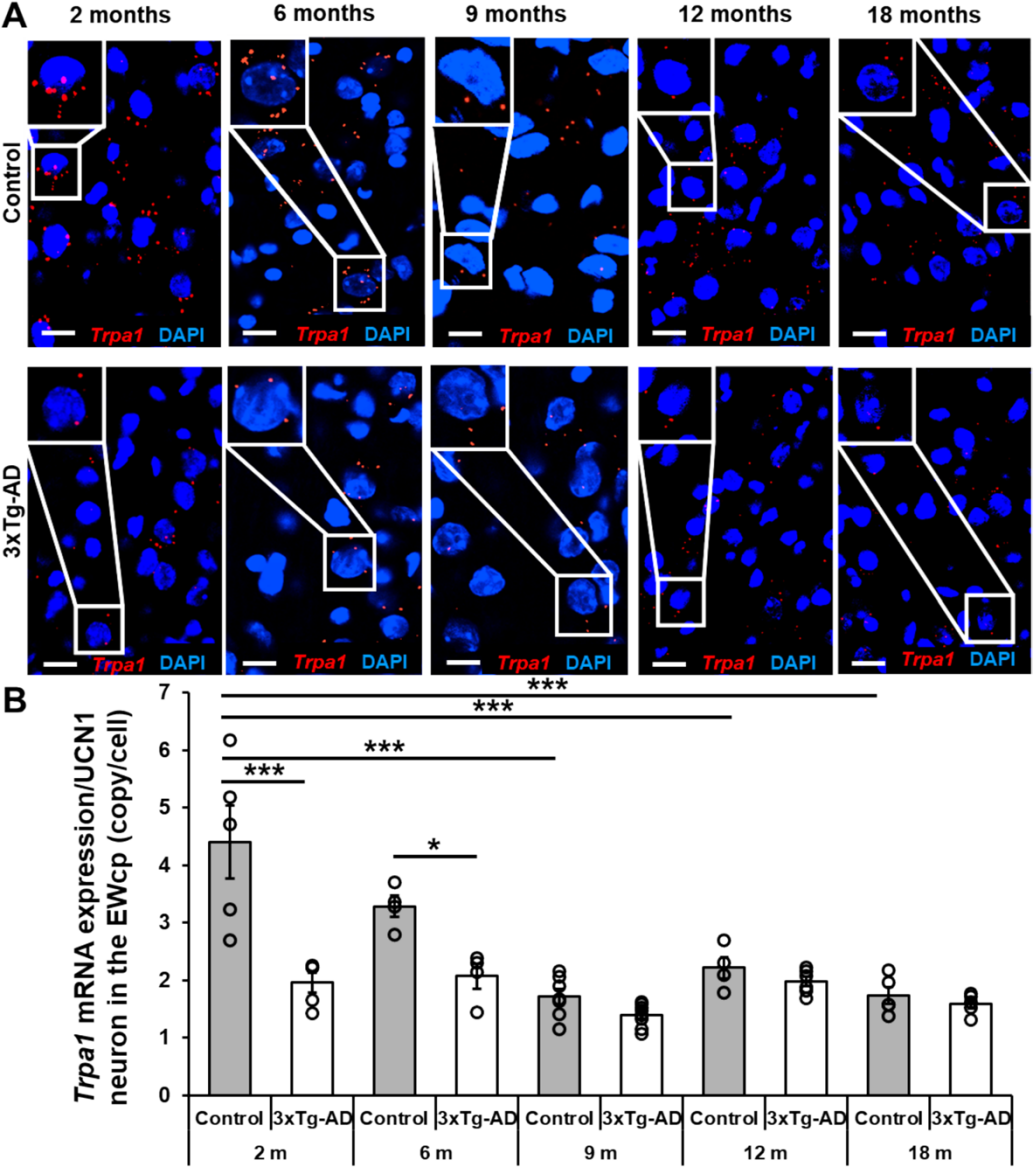
Representative images of the *transient receptor potential ankyrin 1* (*Trpa1*) mRNA expression in the centrally projecting Edinger–Westphal nucleus (EWcp) of control and triple transgenic (3xTg-AD) mice. Red dots represent *Trpa1* mRNA copies, while cell nuclei were counterstained with 4′,6-diamidino-2-phenylindole (DAPI) (blue). Scale bars: 25 μm (**A**). Age-dependent changes in *Trpa1* mRNA copy number in the EWcp of control and 3xTg-AD mice. Two-way ANOVA followed by Tukey’s post hoc test; *p < 0.05 and ***p < 0.001; n = 4–6 (**B**).

We replicated our previous results showing the colocalization of *Trpa1* mRNA copies with the UCN1 immunofluorescence signal. Statistical analysis revealed an age-dependent decrease of the *Trpa1* mRNA expression in control mice. *Trpa1* copy number was significantly higher in the first two (2 and 6 months old) groups compared to their older counterparts (post hoc test *p* < 0.0002 in each comparison of the 2-months-old group, as well as, p_6month/9 month_ < 0.0003; p_6month/12 month_ < 0,11 and p_6month/18 month_ < 0.0008) with a significant main effect of age by ANOVA (F_age_ (4,43) = 21.07; *p* < 0.0001). In contrast, similar age-dependent alterations in *Trpa1* copy number were not observable in the transgenic mice. A permanently lower expression level of *Trpa1* mRNA was associated with significant differences between control and 3xTg-AD animals in the first two age groups (post hoc comparison p_control/3xTg-AD_ < 0.0002 in the 2-months-old and p_control/3xTg-AD_ < 0.04 in the 6-months-old groups) confirmed by the main genotype effect (F_genotype_ (1,43) = 37.69; *p* < 0.0001). The age-dependent reduction of *Trpa1* mRNA expression in the control animals abolished the genotype differences in the older age-groups, supported by the significant interaction between the two variables (F_interaction_ (4,43) = 7.70; *p* < 0.0001) (Fig. 3).

### Age-dependent decrease of taurine/creatine ratio was observed in the hippocampus of male 3xTg-AD AD model without any changes of NAA

We performed MRI-spectroscopy to determine NAA/creatine and taurine/creatine ratios in hippocampus, two biomarkers of brain metabolism.

**Fig. 4:**
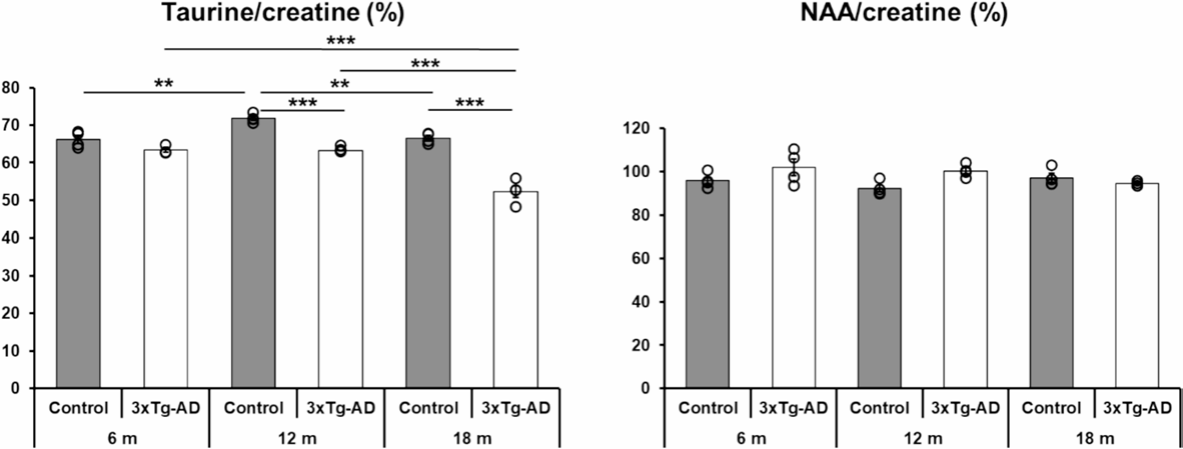
Age- and genotype-dependent changes in taurine/creatine (**A**) and N-acetylaspartate (NAA)/creatine (**B**) ratios of hippocampus in control and triple transgenic (3xTg-AD) mice. Two-way ANOVA followed by Tukey’s post hoc test; *p < 0.05; **p < 0.01; ***p < 0.001 (n = 4).

No initial difference in the taurine/creatine ratio was revealed between the 6 months old groups (*p* = 0.40); however, an age-dependent decrease was observed, with significantly lower values in both 12- and 18-month-old transgenic animals compared to the control counterparts (*p* = 0.0002 in both comparison) supported by the significant main effect of genotype (F_genotype_ (1,17) = 117.61; *p* < 0.0001). Interestingly, the taurine/creatine ratio decreased significantly in the 18-month-old 3xTg-AD animals compared to the younger groups (both p_6month/18 month_ and p_12month/18 month_ = 0.0002). In contrast, no significant difference was observed in taurine/creatine ratio between the 6- and 18-months-old control animals (*p* = 1.00); however, the peak ratio of taurine was detected in the 12-months-old control group (p_6month/12 month_ = 0.005 and p_12month/18mont_ = 0.009) as confirmed by the strong main effect of age (F_age_ (2,17) = 40.87; *p* < 0.0001) together with a significant interaction between the two variables (F_interaction_ (2,17) = 17.60; *p* < 0.0001) by ANOVA (Fig. 4A).

Investigating the changes in the NAA ratio, main genotype effect was significant (F_genotype_ (1,18) = 4.74; *p* = 0.04) with higher levels in 3xTg-AD than controls. However post hoc test did not reveal any significant difference in the NAA/creatine ratio between the two genotypes (*p* > 0.13 in each comparison). In addition, we did not detect any age-dependent alteration in the NAA ratio (F_age_ (2,18) = 1.24; *p* = 0.31; post hoc *p*-value > 0.18 in each comparison) and no significant interaction was established between genotype and age (F_interaction_ (2,18) = 3.52; *p* = 0.051) (Fig. 4B).

## Discussion

The EWcp UCN1 content fluctuated with age in control, while a constant increase was observable in 3xTg-AD animals with a drop at 18 months. The EWcp *Trpa1* mRNA did not follow this trend, it was rather constantly low in transgenes and declined with age in controls. In the hippocampus taurine/creatine ratios were lower in 3xTg-AD than in controls with an age-dependent decrease, while no genotype or age-related changes were revealed in NAA values.

Our previous study suggests that neurodegeneration of urocortinergic EWcp contributes to the mood-related (anxiety, anhedonia and depression-like behavior) symptoms in toxic rat models of Parkinson’s disease. In these paradigms, the loss of UCN1 neurons was accompanied by a parallel decrease in *Ucn1* mRNA expression and increase in peptide content suggesting the inhibited UCN1 release with consequent peptide accumulation and mRNA downregulation^25^.

Since TRPA1 ion channels may enhance the neuropeptide release via calcium-ion influx, the age-related *Trpa1* downregulation of controls may result in a gradual UCN1 peptide retention in the EWcp neurons. In contrast, the low expression of *Trpa1* mRNA in 3xTg-AD mice from the youngest age-group may lead to a higher degree of initial UCN1 retention causing the more intense enhancement of UCN1 peptide content until 12 months of age compared to the controls. This inappropriate release of UCN1 may be involved in the age-related altered stress adaptation and memory decline observed upon aging and in transgenic mice^66–70^.

Substantial evidence demonstrates that UCN1 release influences behavior^17,71–74^, particularly mood, with pronounced effects in the dorsal raphe nucleus (DRN)^42^. UCN1 released from the EWcp can affect the DRN via its direct projection pathway. The DRN plays a critical role in mood regulation, primarily through its serotonergic neurons. Activation of the TRPA1 channels may increase calcium influx, thereby enhancing UCN1 release. Urocortinergic neurons from the EWcp innervate serotonergic cells in the DRN. These cells express both CRFR1 and CRFR2 receptors that exert opposing effects: CRFR1 signaling reduces 5-hydroxitryptamine (5-HT) release, potentially producing anxiogenic effects. In contrast, CRFR2 activation increases 5-HT release, promoting anxiolytic outcomes^75,76^. The above mentioned mechanisms may be involved in mood-related symptoms of the AD.

Beside the stress adaptation, several projection areas of the EWcp/UCN1 neurons are involved in associative memory, memory consolidation and recalling *e.g.*: amygdala^35^, PFC^36^, hippocampus^77^, each of them affected by age-dependent memory decline and AD as well^13,78^. Literature data suggest that 3xTg-AD mice exhibit significant age-related impairment of both working and reference memory compared to the C57Bl/6 genotype as confirmed by increased errors in Morris water maze and radial arm maze tests. Notably, the onset of memory decline manifests earlier in transgenic mice (at 2–4 months of age) as in controls (at 6 months) highlighting a more expressed age-dependent memory impairment in the mouse model of AD^66,68,69^. Considering the neuronal circuits between EWcp and brain areas involved in memory formation—as well as their connection with AD—UCN1 released from EWcp fibers may modulate the memory functions explaining both the age-related alterations in the UCN1 peptide content and the accelerated progression of memory decline in transgenic animals.

Our further morphological analyses revealed more significant age-dependent changes in the UCN1 peptide content of the EWcp in 3xTg-AD AD model. Interestingly, earlier studies have suggested that the TRPA1 ion channel in the EWcp/UCN1 neurons play a role in stress-related disorders both in mice and humans: reduced *Trpa1* mRNA expression was observed in the mouse models of chronic stress and post-traumatic stress disorder (PTSD)^26,61^, moreover in human suicide victims^26^ associated with altered UCN1 turnover. UCN1 belongs to the corticotrophin releasing hormone (CRH) peptide family and has an important role in stress adaptation^16,26,61,72,79–82^. As the TRPA1 ion channel can control neuropeptide release via the regulation of the calcium influx^83–86^, we assume that the decreased *Trpa1* expression contribute to the mood control via UCN1 release from the EWcp. In addition to the fact that based on our previous results we believe that the higher UCN1 density may be due to the inhibited release of the neuropeptide, it is important to mention, that alternative explanations are also possible, including disease-stage-dependent transcriptional regulation, compensatory changes in peptide synthesis or reduced degradation could also account for this pattern of the UCN1 density. Further studies are needed to clarify this interpretation (e.g. *Ucn1* mRNA expression). Interestingly, the AD and age-related memory decline can be associated with stress adaptation disorders (depression and anxiety)^87,88^, moreover, the age-dependent progression of depression and anxiety disorders (*e.g.* generalized anxiety, panic disorder and PTSD) were observed in 3xTg-AD AD mice^89–91^.

The TRPA1 activation may contribute to the acceleration of age-related memory decline and AD^92,93^. Proinflammatory mediators and reactive free radicals may be released during neurodegenerative and neuroinflammatory processes both in AD and in age-associated dementia and are able to activate TRPA1 ion-channels^28,29,31,32,34^ directly contributing to the neurodegeneration in AD^94,95^. The reduced expression of *Trpa1* mRNA of 3xTg-AD mice may be explained by receptor downregulation resulting from the long-lasting agonist action over time^96^.

This may reflect a general pharmacological tolerance mechanism in which prolonged agonist exposure leads to receptor internalization, as observed, for example, with the GnRH receptor^97^, GABA receptor^98^ and opioid receptor^99^. As internalized receptors are no longer required for signaling at the protein level, the cellular demand for newly synthesized TRPA1 channels decreases. Consequently, this reduced requirement may reflected at the transcriptional level as a downregulation of Trpa1 mRNA expression. Thus, lowered *Trpa1* transcript levels can be interpreted as a downstream consequence of receptor internalization-mediated tolerance rather than an independent regulatory event. The age-related decrease in *Trpa1* expression might be a protective response to prevent excessive calcium influx and excitotoxicity, as a compensatory mechanism upon AD progression^100–102^.

We would like to emphasize the translational relevance of our findings, as TRPA1 has also been detected in the urocortinergic cells of the human Edinger–Westphal nucleus^26^, (Al-Omari et al. 2025).^138^

However, via in vivo MRI-spectroscopy, we successfully validated our mouse model at metabolite level, as in the 3xTg-AD mice taurine/creatine ratio of hippocampus showed an age-dependent decline. This metabolite is produced particularly in neurons, it modulates the neuronal function and as free radical scavenger may contribute to neuroprotection^103–108^ decelerating the neurodegeneration upon oxidative stress and neuroinflammation^64,109^. Lower taurine level is associated with hippocampal atrophy in AD, a neuroanatomical change that correlates with the decline in memory and learning abilities^110^. Changes in taurine level may influence the neurodegenerative processes and related cognitive decline via the altered function of TRPA1 ion channels as well. Since taurine depletion exacerbates oxidative stress in AD, we suspect that reduced taurine levels in the hippocampus of old 3xTg-AD animals may activate TRPA1 ion channels in the EW nucleus. Mediators released during taurine-depletion-induced oxidative stress (such as hydrogen peroxide, methylglyoxal, proinflammatory agents) can act as TRPA1 agonists. This could potentially amplify neuroinflammation and neuronal damage^28–34,111^. Interestingly, control animals showed an elevated taurine/creatine ratio by the 12 months of age followed by the reduction of this metabolite to the initial value at the 18 months. This waving alteration in taurine levels observed during physiological aging was reported in previous studies as well^112^. The initial elevation may provide compensation, which counteracts the increased oxidative stress, mitochondrial dysfunction and neuroinflammation supporting neuronal plasticity and moderating early cognitive decline^113^. The delayed decrease of taurine level may result from the reduced endogenous synthesis^114^, impaired cellular transport^115^ and elevated metabolic demand associated with progressive aging^109,116^ leading to depleted cellular storages^110^.

NAA is a biomarker in the central nervous system predominantly found in neurons providing the assessment of neuronal integrity, function and viabilit^117^, whose higher values may indicate healthy, properly functioning neurons, while reduced levels are associated with neuronal loss or dysfunction^118,119^. In our experiment, we did not observe any age-dependent significant differences in the NAA levels in the hippocampus. The possible explanation of these results may be that the early mitochondrial dysfunction of impaired cells and the mild neuronal loss at the initial stages of the disease may lead to increased mitochondrial activity in the surviving, properly functioning cells maintaining the normal NAA level through its elevated synthesis, and even also slightly elevated levels can occur due to increased metabolic demand in 3xTg-AD animals^120–122^. In addition, the impaired oxidative phosphorylation caused by oxidative stress and neuroinflammation may result in a metabolic shift in favor of glycolysis providing adequate ATP levels crucial for the synthesis of NAA as well^123,124^.

### Limitations

One limitation of the study is that only male mice were included. Female mice were excluded to avoid the potential confounding effects of changes related to the hormonal cycle, given that estrogen receptor expression in the Edinger–Westphal nucleus may influence the results^125^. Additionally, the number of animals in the MRI-spectroscopy examination was relatively low due to the high cost of the procedure. However, this sample size is considered acceptable in the literature^126^. Furthermore, RNAscope evaluation is considered a semi-quantitative method. Another limitation of our study is that we have not experimentally verified whether reduced TRPA1 expression directly leads to impaired UCN1 release. As this is a hypothesis it will need to be addressed in future work.

## Conclusion

**Fig. 5:**
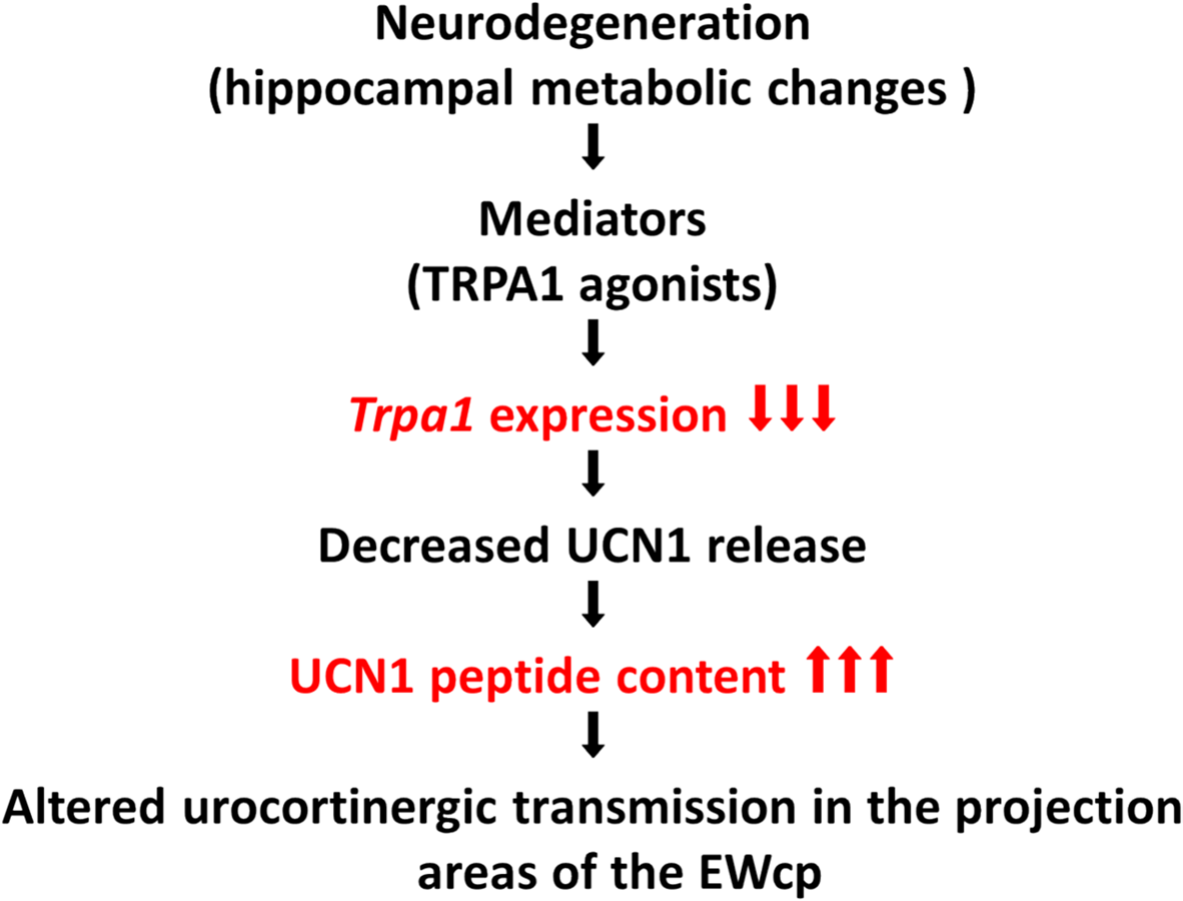
Possible explanation of the results. TRPA1: transient receptor potential ankyrin 1; UCN1: urocortin 1; EWcp: centrally projecting Edinger–Westphal nucleus.

The endogenous mediators released during neurodegeneration may influence the function of urocortinergic EWcp neurons via the age-dependent downregulation of TRPA1 associated with changes in UCN1 peptide content. Further research is required to examine how altered UCN1 signaling contributes to AD-associated mood disorders and memory decline (Fig. 5).

## Data Availability

The datasets used and/or analyzed during the current study are available from the corresponding author upon reasonable request.
